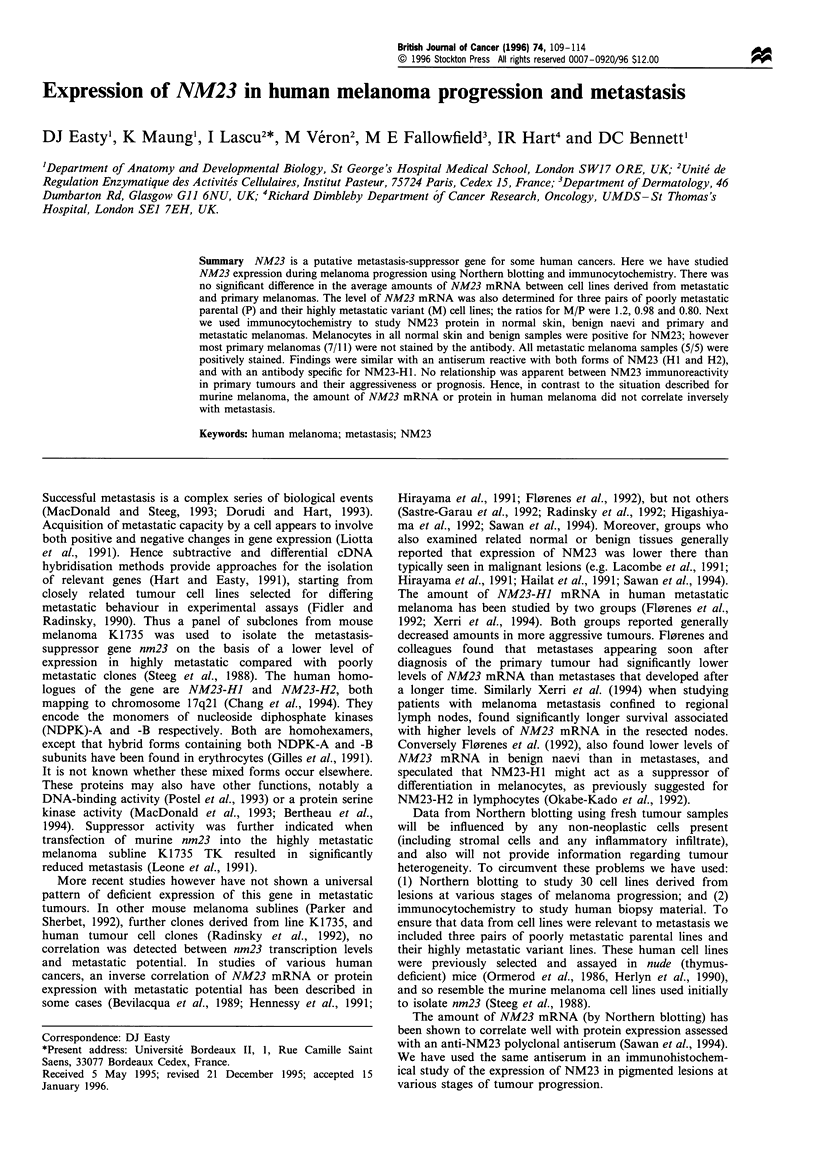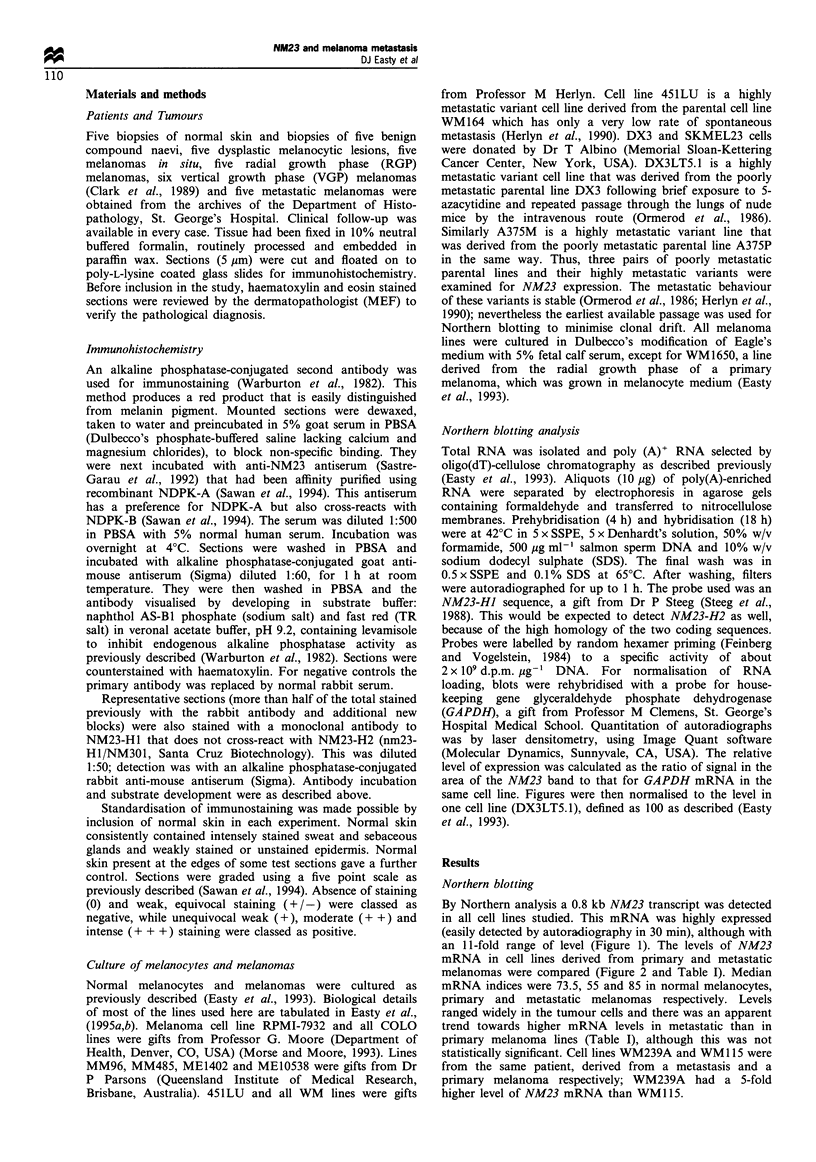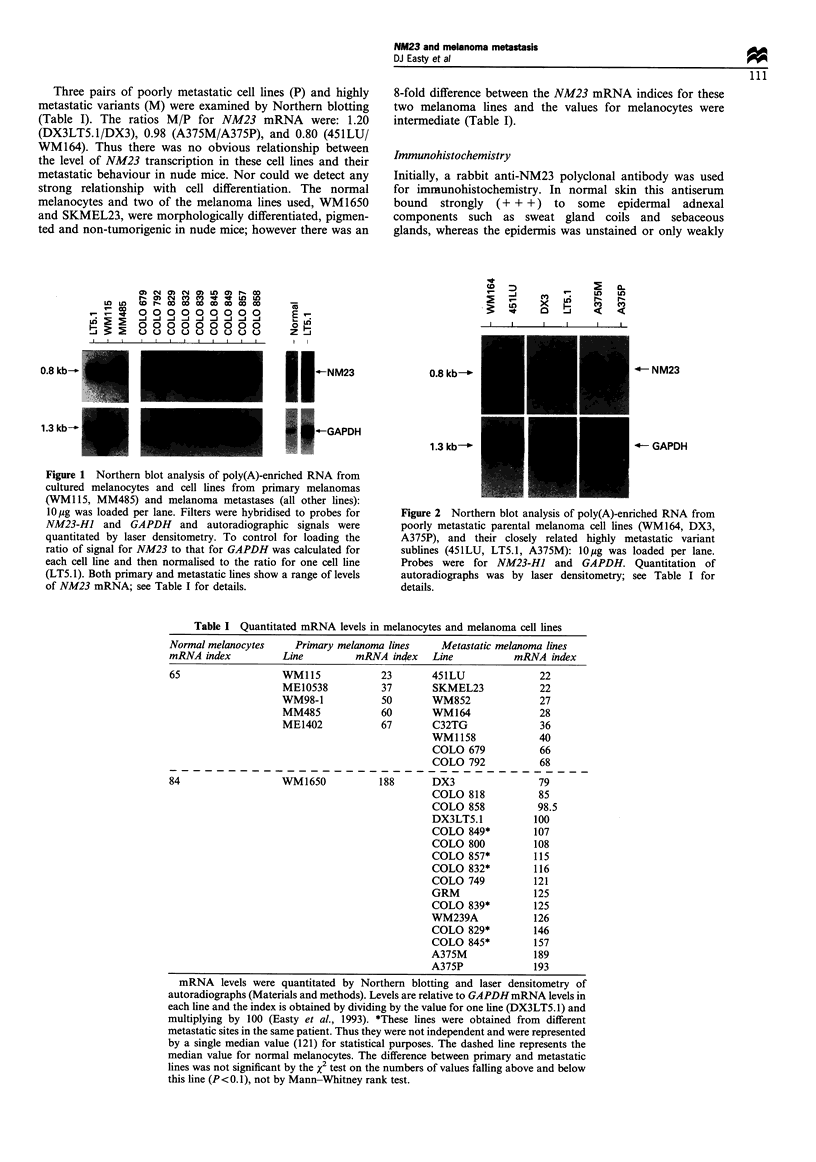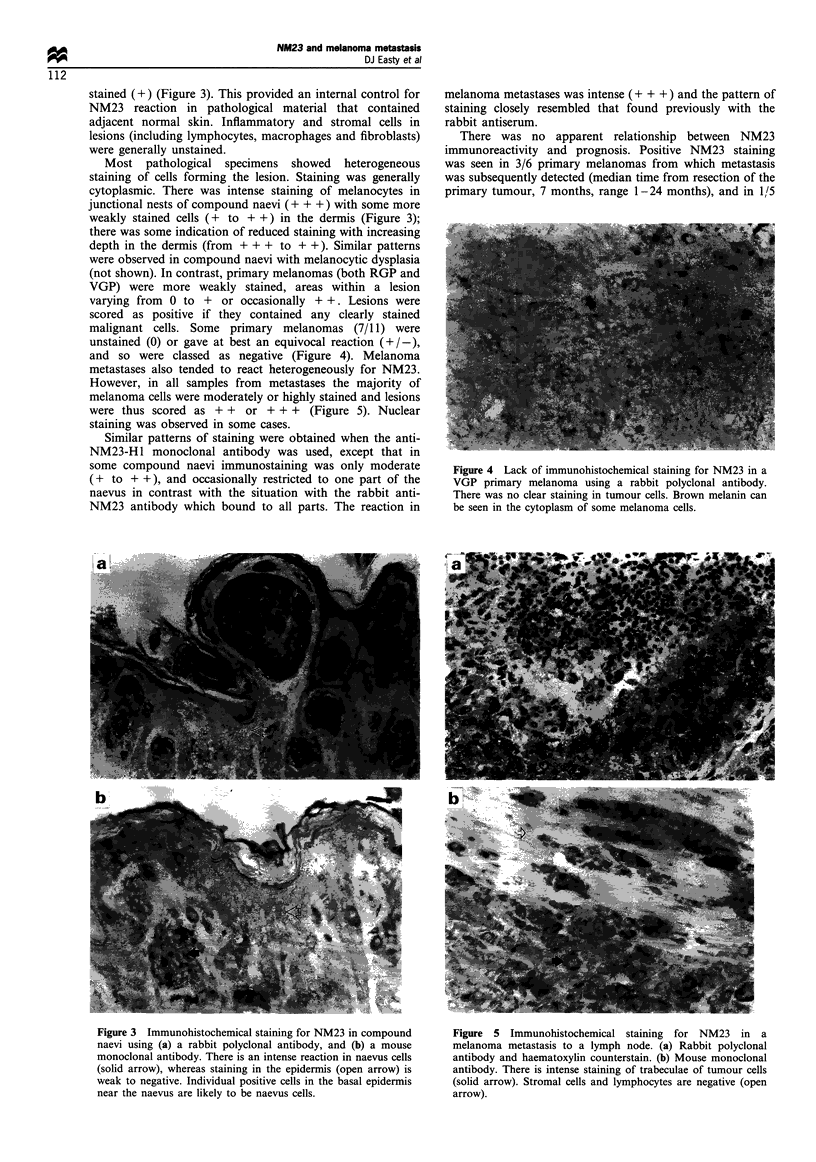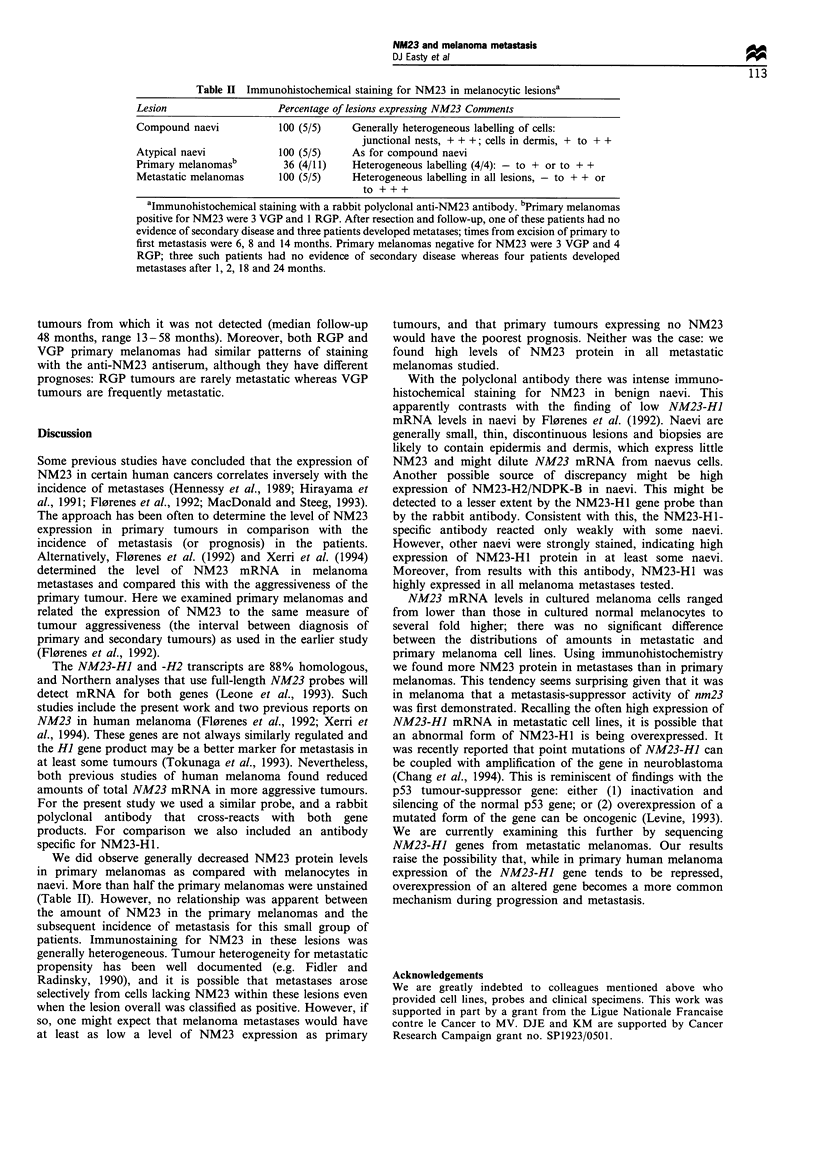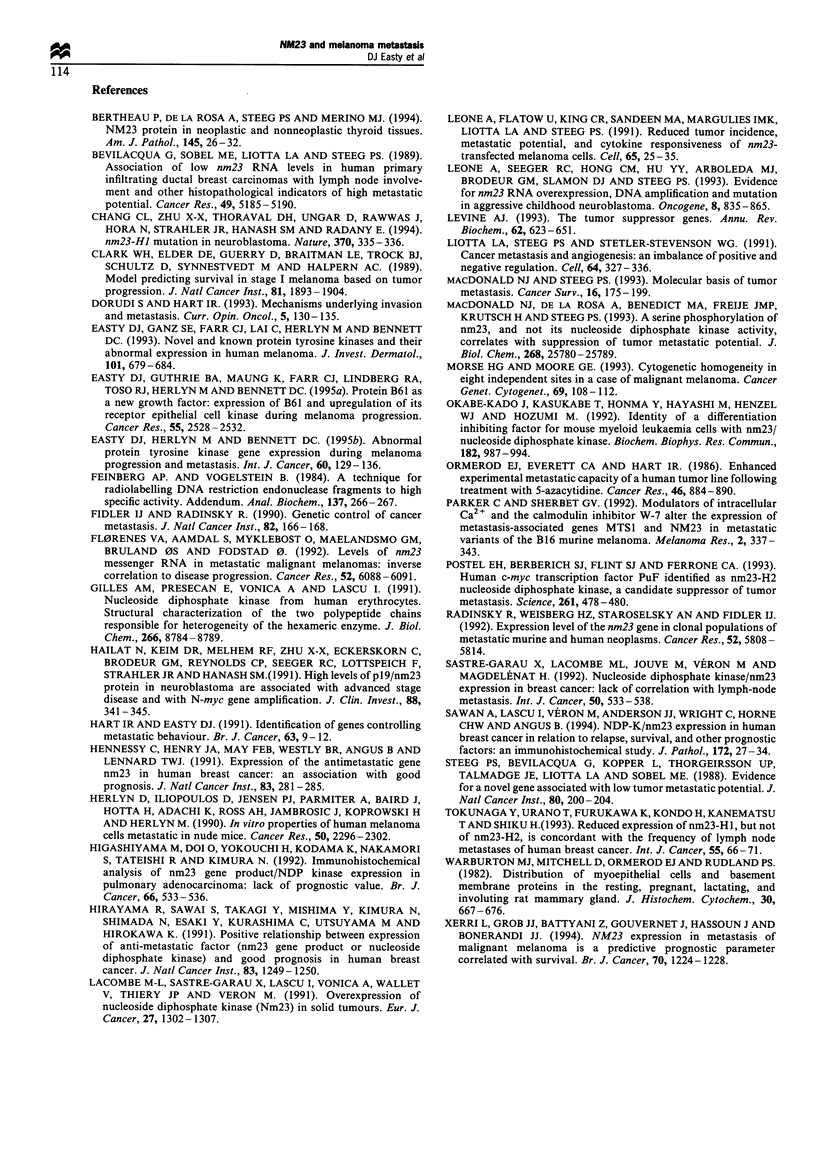# Expression of NM23 in human melanoma progression and metastasis.

**DOI:** 10.1038/bjc.1996.323

**Published:** 1996-07

**Authors:** D. J. Easty, K. Maung, I. Lascu, M. Véron, M. E. Fallowfield, I. R. Hart, D. C. Bennett

**Affiliations:** Department of Anatomy and Developmental Biology, St George's Hospital Medical School, London, UK.

## Abstract

**Images:**


British Journal of Cancer (1996) 74, 109-114
© 1996 Stockton Press All rights reserved 0007-0920/96 $12.00

### Expression of *NM23* in human melanoma progression and metastasis

DJ Easty[1], K Maung[1], I Lascu[2]*, M Véron[2], M E Fallowfield[3], IR Hart[4] and DC Bennett[1]

[1]*Department of Anatomy and Developmental Biology, St George's Hospital Medical School, London SW17 ORE, UK;* [2]*Unité de Regulation Enzymatique des Activités Cellulaires, Institut Pasteur, 75724 Paris, Cedex 15, France;* [3]*Department of Dermatology, 46 Dumbarton Rd, Glasgow G11 6NU, UK;* [4]*Richard Dimbleby Department of Cancer Research, Oncology, UMDS-St Thomas's Hospital, London SE1 7EH, UK.*

**Summary** *NM23* is a putative metastasis-suppressor gene for some human cancers. Here we have studied *NM23* expression during melanoma progression using Northern blotting and immunocytochemistry. There was no significant difference in the average amounts of *NM23* mRNA between cell lines derived from metastatic and primary melanomas. The level of *NM23* mRNA was also determined for three pairs of poorly metastatic parental (P) and their highly metastatic variant (M) cell lines; the ratios for M/P were 1.2, 0.98 and 0.80. Next we used immunocytochemistry to study NM23 protein in normal skin, benign naevi and primary and metastatic melanomas. Melanocytes in all normal skin and benign samples were positive for NM23; however most primary melanomas (7/11) were not stained by the antibody. All metastatic melanoma samples (5/5) were positively stained. Findings were similar with an antiserum reactive with both forms of NM23 (H1 and H2), and with an antibody specific for NM23-H1. No relationship was apparent between NM23 immunoreactivity in primary tumours and their aggressiveness or prognosis. Hence, in contrast to the situation described for murine melanoma, the amount of *NM23* mRNA or protein in human melanoma did not correlate inversely with metastasis.

**Keywords:** human melanoma; metastasis; NM23

Successful metastasis is a complex series of biological events (MacDonald and Steeg, 1993; Dorudi and Hart, 1993). Acquisition of metastatic capacity by a cell appears to involve both positive and negative changes in gene expression (Liotta *et al.*, 1991). Hence subtractive and differential cDNA hybridisation methods provide approaches for the isolation of relevant genes (Hart and Easty, 1991), starting from closely related tumour cell lines selected for differing metastatic behaviour in experimental assays (Fidler and Radinsky, 1990). Thus a panel of subclones from mouse melanoma K1735 was used to isolate the metastasis-suppressor gene *nm23* on the basis of a lower level of expression in highly metastatic compared with poorly metastatic clones (Steeg *et al.*, 1988). The human homologues of the gene are *NM23-H1* and *NM23-H2*, both mapping to chromosome 17q21 (Chang *et al.*, 1994). They encode the monomers of nucleoside diphosphate kinases (NDPK)-A and -B respectively. Both are homohexamers, except that hybrid forms containing both NDPK-A and -B subunits have been found in erythrocytes (Gilles *et al.*, 1991). It is not known whether these mixed forms occur elsewhere. These proteins may also have other functions, notably a DNA-binding activity (Postel *et al.*, 1993) or a protein serine kinase activity (MacDonald *et al.*, 1993; Bertheau *et al.*, 1994). Suppressor activity was further indicated when transfection of murine *nm23* into the highly metastatic melanoma subline K1735 TK resulted in significantly reduced metastasis (Leone *et al.*, 1991).

More recent studies however have not shown a universal pattern of deficient expression of this gene in metastatic tumours. In other mouse melanoma sublines (Parker and Sherbet, 1992), further clones derived from line K1735, and human tumour cell clones (Radinsky *et al.*, 1992), no correlation was detected between *nm23* transcription levels and metastatic potential. In studies of various human cancers, an inverse correlation of *NM23* mRNA or protein expression with metastatic potential has been described in some cases (Bevilacqua *et al.*, 1989; Hennessy *et al.*, 1991; Hirayama *et al.*, 1991; Flørenes *et al.*, 1992), but not others (Sastre-Garau *et al.*, 1992; Radinsky *et al.*, 1992; Higashiyama *et al.*, 1992; Sawan *et al.*, 1994). Moreover, groups who also examined related normal or benign tissues generally reported that expression of NM23 was lower there than typically seen in malignant lesions (e.g. Lacombe *et al.*, 1991; Hirayama *et al.*, 1991; Hailat *et al.*, 1991; Sawan *et al.*, 1994). The amount of *NM23-H1* mRNA in human metastatic melanoma has been studied by two groups (Flørenes *et al.*, 1992; Xerri *et al.*, 1994). Both groups reported generally decreased amounts in more aggressive tumours. Flørenes and colleagues found that metastases appearing soon after diagnosis of the primary tumour had significantly lower levels of *NM23* mRNA than metastases that developed after a longer time. Similarly Xerri *et al.* (1994) when studying patients with melanoma metastasis confined to regional lymph nodes, found significantly longer survival associated with higher levels of *NM23* mRNA in the resected nodes. Conversely Flørenes *et al.* (1992), also found lower levels of *NM23* mRNA in benign naevi than in metastases, and speculated that NM23-H1 might act as a suppressor of differentiation in melanocytes, as previously suggested for NM23-H2 in lymphocytes (Okabe-Kado *et al.*, 1992).

Data from Northern blotting using fresh tumour samples will be influenced by any non-neoplastic cells present (including stromal cells and any inflammatory infiltrate), and also will not provide information regarding tumour heterogeneity. To circumvent these problems we have used: (1) Northern blotting to study 30 cell lines derived from lesions at various stages of melanoma progression; and (2) immunocytochemistry to study human biopsy material. To ensure that data from cell lines were relevant to metastasis we included three pairs of poorly metastatic parental lines and their highly metastatic variant lines. These human cell lines were previously selected and assayed in *nude* (thymus-deficient) mice (Ormerod *et al.*, 1986, Herlyn *et al.*, 1990), and so resemble the murine melanoma cell lines used initially to isolate *nm23* (Steeg *et al.*, 1988).

The amount of *NM23* mRNA (by Northern blotting) has been shown to correlate well with protein expression assessed with an anti-NM23 polyclonal antiserum (Sawan *et al.*, 1994). We have used the same antiserum in an immunohistochemical study of the expression of NM23 in pigmented lesions at various stages of tumour progression.

Correspondence: DJ Easty
*Present address: Université Bordeaux II, 1, Rue Camille Saint Saens, 33077 Bordeaux Cedex, France.
Received 5 May 1995; revised 21 December 1995; accepted 15 January 1996.

### Materials and methods

*Patients and Tumours*

Five biopsies of normal skin and biopsies of five benign compound naevi, five dysplastic melanocytic lesions, five melanomas *in situ*, five radial growth phase (RGP) melanomas, six vertical growth phase (VGP) melanomas (Clark *et al.*, 1989) and five metastatic melanomas were obtained from the archives of the Department of Histopathology, St. George's Hospital. Clinical follow-up was available in every case. Tissue had been fixed in 10% neutral buffered formalin, routinely processed and embedded in paraffin wax. Sections (5 $\mu$m) were cut and floated on to poly-L-lysine coated glass slides for immunohistochemistry. Before inclusion in the study, haematoxylin and eosin stained sections were reviewed by the dermatopathologist (MEF) to verify the pathological diagnosis.

*Immunohistochemistry*

An alkaline phosphatase-conjugated second antibody was used for immunostaining (Warburton *et al.*, 1982). This method produces a red product that is easily distinguished from melanin pigment. Mounted sections were dewaxed, taken to water and preincubated in 5% goat serum in PBSA (Dulbecco's phosphate-buffered saline lacking calcium and magnesium chlorides), to block non-specific binding. They were next incubated with anti-NM23 antiserum (Sastre-Garau *et al.*, 1992) that had been affinity purified using recombinant NDPK-A (Sawan *et al.*, 1994). This antiserum has a preference for NDPK-A but also cross-reacts with NDPK-B (Sawan *et al.*, 1994). The serum was diluted 1:500 in PBSA with 5% normal human serum. Incubation was overnight at 4°C. Sections were washed in PBSA and incubated with alkaline phosphatase-conjugated goat anti-mouse antiserum (Sigma) diluted 1:60, for 1 h at room temperature. They were then washed in PBSA and the antibody visualised by developing in substrate buffer: naphthol AS-B1 phosphate (sodium salt) and fast red (TR salt) in veronal acetate buffer, pH 9.2, containing levamisole to inhibit endogenous alkaline phosphatase activity as previously described (Warburton *et al.*, 1982). Sections were counterstained with haematoxylin. For negative controls the primary antibody was replaced by normal rabbit serum.

Representative sections (more than half of the total stained previously with the rabbit antibody and additional new blocks) were also stained with a monoclonal antibody to NM23-H1 that does not cross-react with NM23-H2 (nm23-H1/NM301, Santa Cruz Biotechnology). This was diluted 1:50; detection was with an alkaline phosphatase-conjugated rabbit anti-mouse antiserum (Sigma). Antibody incubation and substrate development were as described above.

Standardisation of immunostaining was made possible by inclusion of normal skin in each experiment. Normal skin consistently contained intensely stained sweat and sebaceous glands and weakly stained or unstained epidermis. Normal skin present at the edges of some test sections gave a further control. Sections were graded using a five point scale as previously described (Sawan *et al.*, 1994). Absence of staining (0) and weak, equivocal staining (+/−) were classed as negative, while unequivocal weak (+), moderate (+ +) and intense (+ + +) staining were classed as positive.

*Culture of melanocytes and melanomas*

Normal melanocytes and melanomas were cultured as previously described (Easty *et al.*, 1993). Biological details of most of the lines used here are tabulated in Easty *et al.*, (1995*a*,*b*). Melanoma cell line RPMI-7932 and all COLO lines were gifts from Professor G. Moore (Department of Health, Denver, CO, USA) (Morse and Moore, 1993). Lines MM96, MM485, ME1402 and ME10538 were gifts from Dr P Parsons (Queensland Institute of Medical Research, Brisbane, Australia). 451LU and all WM lines were gifts from Professor M Herlyn. Cell line 451LU is a highly metastatic variant cell line derived from the parental cell line WM164 which has only a very low rate of spontaneous metastasis (Herlyn *et al.*, 1990). DX3 and SKMEL23 cells were donated by Dr T Albino (Memorial Sloan-Kettering Cancer Center, New York, USA). DX3LT5.1 is a highly metastatic variant cell line that was derived from the poorly metastatic parental line DX3 following brief exposure to 5-azacytidine and repeated passage through the lungs of nude mice by the intravenous route (Ormerod *et al.*, 1986). Similarly A375M is a highly metastatic variant line that was derived from the poorly metastatic parental line A375P in the same way. Thus, three pairs of poorly metastatic parental lines and their highly metastatic variants were examined for *NM23* expression. The metastatic behaviour of these variants is stable (Ormerod *et al.*, 1986; Herlyn *et al.*, 1990); nevertheless the earliest available passage was used for Northern blotting to minimise clonal drift. All melanoma lines were cultured in Dulbecco's modification of Eagle's medium with 5% fetal calf serum, except for WM1650, a line derived from the radial growth phase of a primary melanoma, which was grown in melanocyte medium (Easty *et al.*, 1993).

*Northern blotting analysis*

Total RNA was isolated and poly $(A)^+$ RNA selected by oligo(dT)-cellulose chromatography as described previously (Easty *et al.*, 1993). Aliquots (10 $\mu$g) of poly(A)-enriched RNA were separated by electrophoresis in agarose gels containing formaldehyde and transferred to nitrocellulose membranes. Prehybridisation (4 h) and hybridisation (18 h) were at 42°C in $5\times$SSPE, $5\times$Denhardt's solution, 50% w/v formamide, 500 $\mu g\ ml^{-1}$ salmon sperm DNA and 10% w/v sodium dodecyl sulphate (SDS). The final wash was in $0.5\times$SSPE and 0.1% SDS at 65°C. After washing, filters were autoradiographed for up to 1 h. The probe used was an *NM23-H1* sequence, a gift from Dr P Steeg (Steeg *et al.*, 1988). This would be expected to detect *NM23-H2* as well, because of the high homology of the two coding sequences. Probes were labelled by random hexamer priming (Feinberg and Vogelstein, 1984) to a specific activity of about $2\times10^9$ d.p.m. $\mu g^{-1}$ DNA. For normalisation of RNA loading, blots were rehybridised with a probe for house-keeping gene glyceraldehyde phosphate dehydrogenase (*GAPDH*), a gift from Professor M Clemens, St. George's Hospital Medical School. Quantitation of autoradiographs was by laser densitometry, using Image Quant software (Molecular Dynamics, Sunnyvale, CA, USA). The relative level of expression was calculated as the ratio of signal in the area of the *NM23* band to that for *GAPDH* mRNA in the same cell line. Figures were then normalised to the level in one cell line (DX3LT5.1), defined as 100 as described (Easty *et al.*, 1993).

### Results

*Northern blotting*

By Northern analysis a 0.8 kb *NM23* transcript was detected in all cell lines studied. This mRNA was highly expressed (easily detected by autoradiography in 30 min), although with an 11-fold range of level (Figure 1). The levels of *NM23* mRNA in cell lines derived from primary and metastatic melanomas were compared (Figure 2 and Table I). Median mRNA indices were 73.5, 55 and 85 in normal melanocytes, primary and metastatic melanomas respectively. Levels ranged widely in the tumour cells and there was an apparent trend towards higher mRNA levels in metastatic than in primary melanoma lines (Table I), although this was not statistically significant. Cell lines WM239A and WM115 were from the same patient, derived from a metastasis and a primary melanoma respectively; WM239A had a 5-fold higher level of *NM23* mRNA than WM115.

Three pairs of poorly metastatic cell lines (P) and highly metastatic variants (M) were examined by Northern blotting (Table I). The ratios M/P for *NM23* mRNA were: 1.20 (DX3LT5.1/DX3), 0.98 (A375M/A375P), and 0.80 (451LU/WM164). Thus there was no obvious relationship between the level of *NM23* transcription in these cell lines and their metastatic behaviour in nude mice. Nor could we detect any strong relationship with cell differentiation. The normal melanocytes and two of the melanoma lines used, WM1650 and SKMEL23, were morphologically differentiated, pigmented and non-tumorigenic in nude mice; however there was an 8-fold difference between the *NM23* mRNA indices for these two melanoma lines and the values for melanocytes were intermediate (Table I).

*Immunohistochemistry*

Initially, a rabbit anti-NM23 polyclonal antibody was used for immunohistochemistry. In normal skin this antiserum bound strongly (+++) to some epidermal adnexal components such as sweat gland coils and sebaceous glands, whereas the epidermis was unstained or only weakly

**Figure 1** Northern blot analysis of poly(A)-enriched RNA from cultured melanocytes and cell lines from primary melanomas (WM115, MM485) and melanoma metastases (all other lines): 10 μg was loaded per lane. Filters were hybridised to probes for *NM23-H1* and *GAPDH* and autoradiographic signals were quantitated by laser densitometry. To control for loading the ratio of signal for *NM23* to that for *GAPDH* was calculated for each cell line and then normalised to the ratio for one cell line (LT5.1). Both primary and metastatic lines show a range of levels of *NM23* mRNA; see Table I for details.

**Figure 2** Northern blot analysis of poly(A)-enriched RNA from poorly metastatic parental melanoma cell lines (WM164, DX3, A375P), and their closely related highly metastatic variant sublines (451LU, LT5.1, A375M): 10 μg was loaded per lane. Probes were for *NM23-H1* and *GAPDH*. Quantitation of autoradiographs was by laser densitometry; see Table I for details.

**Table I** Quantitated mRNA levels in melanocytes and melanoma cell lines

| *Normal melanocytes* | *Primary melanoma lines* | | *Metastatic melanoma lines* | |
|---|---|---|---|---|
| *mRNA index* | *Line* | *mRNA index* | *Line* | *mRNA index* |
| 65 | WM115 | 23 | 451LU | 22 |
| | ME10538 | 37 | SKMEL23 | 22 |
| | WM98-1 | 50 | WM852 | 27 |
| | MM485 | 60 | WM164 | 28 |
| | ME1402 | 67 | C32TG | 36 |
| | | | WM1158 | 40 |
| | | | COLO 679 | 66 |
| | | | COLO 792 | 68 |
| 84 | WM1650 | 188 | DX3 | 79 |
| | | | COLO 818 | 85 |
| | | | COLO 858 | 98.5 |
| | | | DX3LT5.1 | 100 |
| | | | COLO 849* | 107 |
| | | | COLO 800 | 108 |
| | | | COLO 857* | 115 |
| | | | COLO 832* | 116 |
| | | | COLO 749 | 121 |
| | | | GRM | 125 |
| | | | COLO 839* | 125 |
| | | | WM239A | 126 |
| | | | COLO 829* | 146 |
| | | | COLO 845* | 157 |
| | | | A375M | 189 |
| | | | A375P | 193 |

mRNA levels were quantitated by Northern blotting and laser densitometry of autoradiographs (Materials and methods). Levels are relative to *GAPDH* mRNA levels in each line and the index is obtained by dividing by the value for one line (DX3LT5.1) and multiplying by 100 (Easty *et al.*, 1993). *These lines were obtained from different metastatic sites in the same patient. Thus they were not independent and were represented by a single median value (121) for statistical purposes. The dashed line represents the median value for normal melanocytes. The difference between primary and metastatic lines was not significant by the $\chi^2$ test on the numbers of values falling above and below this line ($P<0.1$), not by Mann–Whitney rank test.

stained (+) (Figure 3). This provided an internal control for NM23 reaction in pathological material that contained adjacent normal skin. Inflammatory and stromal cells in lesions (including lymphocytes, macrophages and fibroblasts) were generally unstained.

Most pathological specimens showed heterogeneous staining of cells forming the lesion. Staining was generally cytoplasmic. There was intense staining of melanocytes in junctional nests of compound naevi (+ + +) with some more weakly stained cells (+ to + +) in the dermis (Figure 3); there was some indication of reduced staining with increasing depth in the dermis (from + + + to + +). Similar patterns were observed in compound naevi with melanocytic dysplasia (not shown). In contrast, primary melanomas (both RGP and VGP) were more weakly stained, areas within a lesion varying from 0 to + or occasionally + +. Lesions were scored as positive if they contained any clearly stained malignant cells. Some primary melanomas (7/11) were unstained (0) or gave at best an equivocal reaction (+/−), and so were classed as negative (Figure 4). Melanoma metastases also tended to react heterogeneously for NM23. However, in all samples from metastases the majority of melanoma cells were moderately or highly stained and lesions were thus scored as + + or + + + (Figure 5). Nuclear staining was observed in some cases.

Similar patterns of staining were obtained when the anti-NM23-H1 monoclonal antibody was used, except that in some compound naevi immunostaining was only moderate (+ to + +), and occasionally restricted to one part of the naevus in contrast with the situation with the rabbit anti-NM23 antibody which bound to all parts. The reaction in melanoma metastases was intense (+ + +) and the pattern of staining closely resembled that found previously with the rabbit antiserum.

There was no apparent relationship between NM23 immunoreactivity and prognosis. Positive NM23 staining was seen in 3/6 primary melanomas from which metastasis was subsequently detected (median time from resection of the primary tumour, 7 months, range 1–24 months), and in 1/5

**Figure 4** Lack of immunohistochemical staining for NM23 in a VGP primary melanoma using a rabbit polyclonal antibody. There was no clear staining in tumour cells. Brown melanin can be seen in the cytoplasm of some melanoma cells.

**Figure 3** Immunohistochemical staining for NM23 in compound naevi using (**a**) a rabbit polyclonal antibody, and (**b**) a mouse monoclonal antibody. There is an intense reaction in naevus cells (solid arrow), whereas staining in the epidermis (open arrow) is weak to negative. Individual positive cells in the basal epidermis near the naevus are likely to be naevus cells.

**Figure 5** Immunohistochemical staining for NM23 in a melanoma metastasis to a lymph node. (**a**) Rabbit polyclonal antibody and haematoxylin counterstain. (**b**) Mouse monoclonal antibody. There is intense staining of trabeculae of tumour cells (solid arrow). Stromal cells and lymphocytes are negative (open arrow).

**Table II** Immunohistochemical staining for NM23 in melanocytic lesions[a]

| *Lesion* | *Percentage of lesions expressing NM23* | *Comments* |
|---|---|---|
| Compound naevi | 100 (5/5) | Generally heterogeneous labelling of cells: junctional nests, + + +; cells in dermis, + to + + |
| Atypical naevi | 100 (5/5) | As for compound naevi |
| Primary melanomas[b] | 36 (4/11) | Heterogeneous labelling (4/4): − to + or to + + |
| Metastatic melanomas | 100 (5/5) | Heterogeneous labelling in all lesions, − to + + or to + + + |

[a]Immunohistochemical staining with a rabbit polyclonal anti-NM23 antibody. [b]Primary melanomas positive for NM23 were 3 VGP and 1 RGP. After resection and follow-up, one of these patients had no evidence of secondary disease and three patients developed metatases; times from excision of primary to first metastasis were 6, 8 and 14 months. Primary melanomas negative for NM23 were 3 VGP and 4 RGP; three such patients had no evidence of secondary disease whereas four patients developed metastases after 1, 2, 18 and 24 months.

tumours from which it was not detected (median follow-up 48 months, range 13–58 months). Moreover, both RGP and VGP primary melanomas had similar patterns of staining with the anti-NM23 antiserum, although they have different prognoses: RGP tumours are rarely metastatic whereas VGP tumours are frequently metastatic.

### Discussion

Some previous studies have concluded that the expression of NM23 in certain human cancers correlates inversely with the incidence of metastases (Hennessy *et al.*, 1989; Hirayama *et al.*, 1991; Flørenes *et al.*, 1992; MacDonald and Steeg, 1993). The approach has been often to determine the level of NM23 expression in primary tumours in comparison with the incidence of metastasis (or prognosis) in the patients. Alternatively, Flørenes *et al.* (1992) and Xerri *et al.* (1994) determined the level of NM23 mRNA in melanoma metastases and compared this with the aggressiveness of the primary tumour. Here we examined primary melanomas and related the expression of NM23 to the same measure of tumour aggressiveness (the interval between diagnosis of primary and secondary tumours) as used in the earlier study (Flørenes *et al.*, 1992).

The *NM23-H1* and *-H2* transcripts are 88% homologous, and Northern analyses that use full-length *NM23* probes will detect mRNA for both genes (Leone *et al.*, 1993). Such studies include the present work and two previous reports on *NM23* in human melanoma (Flørenes *et al.*, 1992; Xerri *et al.*, 1994). These genes are not always similarly regulated and the *H1* gene product may be a better marker for metastasis in at least some tumours (Tokunaga *et al.*, 1993). Nevertheless, both previous studies of human melanoma found reduced amounts of total *NM23* mRNA in more aggressive tumours. For the present study we used a similar probe, and a rabbit polyclonal antibody that cross-reacts with both gene products. For comparison we also included an antibody specific for NM23-H1.

We did observe generally decreased NM23 protein levels in primary melanomas as compared with melanocytes in naevi. More than half the primary melanomas were unstained (Table II). However, no relationship was apparent between the amount of NM23 in the primary melanomas and the subsequent incidence of metastasis for this small group of patients. Immunostaining for NM23 in these lesions was generally heterogeneous. Tumour heterogeneity for metastatic propensity has been well documented (e.g. Fidler and Radinsky, 1990), and it is possible that metastases arose selectively from cells lacking NM23 within these lesions even when the lesion overall was classified as positive. However, if so, one might expect that melanoma metastases would have at least as low a level of NM23 expression as primary tumours, and that primary tumours expressing no NM23 would have the poorest prognosis. Neither was the case: we found high levels of NM23 protein in all metastatic melanomas studied.

With the polyclonal antibody there was intense immunohistochemical staining for NM23 in benign naevi. This apparently contrasts with the finding of low *NM23-H1* mRNA levels in naevi by Flørenes *et al.* (1992). Naevi are generally small, thin, discontinuous lesions and biopsies are likely to contain epidermis and dermis, which express little NM23 and might dilute *NM23* mRNA from naevus cells. Another possible source of discrepancy might be high expression of NM23-H2/NDPK-B in naevi. This might be detected to a lesser extent by the NM23-H1 gene probe than by the rabbit antibody. Consistent with this, the NM23-H1-specific antibody reacted only weakly with some naevi. However, other naevi were strongly stained, indicating high expression of NM23-H1 protein in at least some naevi. Moreover, from results with this antibody, NM23-H1 was highly expressed in all melanoma metastases tested.

*NM23* mRNA levels in cultured melanoma cells ranged from lower than those in cultured normal melanocytes to several fold higher; there was no significant difference between the distributions of amounts in metastatic and primary melanoma cell lines. Using immunohistochemistry we found more NM23 protein in metastases than in primary melanomas. This tendency seems surprising given that it was in melanoma that a metastasis-suppressor activity of *nm23* was first demonstrated. Recalling the often high expression of *NM23-H1* mRNA in metastatic cell lines, it is possible that an abnormal form of NM23-H1 is being overexpressed. It was recently reported that point mutations of *NM23-H1* can be coupled with amplification of the gene in neuroblastoma (Chang *et al.*, 1994). This is reminiscent of findings with the p53 tumour-suppressor gene: either (1) inactivation and silencing of the normal p53 gene; or (2) overexpression of a mutated form of the gene can be oncogenic (Levine, 1993). We are currently examining this further by sequencing *NM23-H1* genes from metastatic melanomas. Our results raise the possibility that, while in primary human melanoma expression of the *NM23-H1* gene tends to be repressed, overexpression of an altered gene becomes a more common mechanism during progression and metastasis.

**Acknowledgements**
We are greatly indebted to colleagues mentioned above who provided cell lines, probes and clinical specimens. This work was supported in part by a grant from the Ligue Nationale Francaise contre le Cancer to MV. DJE and KM are supported by Cancer Research Campaign grant no. SP1923/0501.

### References

BERTHEAU P, DE LA ROSA A, STEEG PS AND MERINO MJ. (1994). NM23 protein in neoplastic and nonneoplastic thyroid tissues. *Am. J. Pathol.*, **145**, 26-32.

BEVILACQUA G, SOBEL ME, LIOTTA LA AND STEEG PS. (1989). Association of low *nm23* RNA levels in human primary infiltrating ductal breast carcinomas with lymph node involvement and other histopathological indicators of high metastatic potential. *Cancer Res.*, **49**, 5185-5190.

CHANG CL, ZHU X-X, THORAVAL DH, UNGAR D, RAWWAS J, HORA N, STRAHLER JR, HANASH SM AND RADANY E. (1994). *nm23-H1* mutation in neuroblastoma. *Nature*, **370**, 335-336.

CLARK WH, ELDER DE, GUERRY D, BRAITMAN LE, TROCK BJ, SCHULTZ D, SYNNESTVEDT M AND HALPERN AC. (1989). Model predicting survival in stage I melanoma based on tumor progression. *J. Natl Cancer Inst.*, **81**, 1893-1904.

DORUDI S AND HART IR. (1993). Mechanisms underlying invasion and metastasis. *Curr. Opin. Oncol.*, **5**, 130-135.

EASTY DJ, GANZ SE, FARR CJ, LAI C, HERLYN M AND BENNETT DC. (1993). Novel and known protein tyrosine kinases and their abnormal expression in human melanoma. *J. Invest. Dermatol.*, **101**, 679-684.

EASTY DJ, GUTHRIE BA, MAUNG K, FARR CJ, LINDBERG RA, TOSO RJ, HERLYN M AND BENNETT DC. (1995*a*). Protein B61 as a new growth factor: expression of B61 and upregulation of its receptor epithelial cell kinase during melanoma progression. *Cancer Res.*, **55**, 2528-2532.

EASTY DJ, HERLYN M AND BENNETT DC. (1995*b*). Abnormal protein tyrosine kinase gene expression during melanoma progression and metastasis. *Int. J. Cancer*, **60**, 129-136.

FEINBERG AP. AND VOGELSTEIN B. (1984). A technique for radiolabelling DNA restriction endonuclease fragments to high specific activity. Addendum. *Anal. Biochem.*, **137**, 266-267.

FIDLER IJ AND RADINSKY R. (1990). Genetic control of cancer metastasis. *J. Natl Cancer Inst.*, **82**, 166-168.

FLØRENES VA, AAMDAL S, MYKLEBOST O, MAELANDSMO GM, BRULAND ØS AND FODSTAD Ø. (1992). Levels of *nm23* messenger RNA in metastatic malignant melanomas: inverse correlation to disease progression. *Cancer Res.*, **52**, 6088-6091.

GILLES AM, PRESECAN E, VONICA A AND LASCU I. (1991). Nucleoside diphosphate kinase from human erythrocytes. Structural characterization of the two polypeptide chains responsible for heterogeneity of the hexameric enzyme. *J. Biol. Chem.*, **266**, 8784-8789.

HAILAT N, KEIM DR, MELHEM RF, ZHU X-X, ECKERSKORN C, BRODEUR GM, REYNOLDS CP, SEEGER RC, LOTTSPEICH F, STRAHLER JR AND HANASH SM.(1991). High levels of p19/nm23 protein in neuroblastoma are associated with advanced stage disease and with N-*myc* gene amplification. *J. Clin. Invest.*, **88**, 341-345.

HART IR AND EASTY DJ. (1991). Identification of genes controlling metastatic behaviour. *Br. J. Cancer*, **63**, 9-12.

HENNESSY C, HENRY JA, MAY FEB, WESTLY BR, ANGUS B AND LENNARD TWJ. (1991). Expression of the antimetastatic gene nm23 in human breast cancer: an association with good prognosis. *J. Natl Cancer Inst.*, **83**, 281-285.

HERLYN D, ILIOPOULOS D, JENSEN PJ, PARMITER A, BAIRD J, HOTTA H, ADACHI K, ROSS AH, JAMBROSIC J, KOPROWSKI H AND HERLYN M. (1990). *In vitro* properties of human melanoma cells metastatic in nude mice. *Cancer Res.*, **50**, 2296-2302.

HIGASHIYAMA M, DOI O, YOKOUCHI H, KODAMA K, NAKAMORI S, TATEISHI R AND KIMURA N. (1992). Immunohistochemical analysis of nm23 gene product/NDP kinase expression in pulmonary adenocarcinoma: lack of prognostic value. *Br. J. Cancer*, **66**, 533-536.

HIRAYAMA R, SAWAI S, TAKAGI Y, MISHIMA Y, KIMURA N, SHIMADA N, ESAKI Y, KURASHIMA C, UTSUYAMA M AND HIROKAWA K. (1991). Positive relationship between expression of anti-metastatic factor (nm23 gene product or nucleoside diphosphate kinase) and good prognosis in human breast cancer. *J. Natl Cancer Inst.*, **83**, 1249-1250.

LACOMBE M-L, SASTRE-GARAU X, LASCU I, VONICA A, WALLET V, THIERY JP AND VERON M. (1991). Overexpression of nucleoside diphosphate kinase (Nm23) in solid tumours. *Eur. J. Cancer*, **27**, 1302-1307.

LEONE A, FLATOW U, KING CR, SANDEEN MA, MARGULIES IMK, LIOTTA LA AND STEEG PS. (1991). Reduced tumor incidence, metastatic potential, and cytokine responsiveness of *nm23*-transfected melanoma cells. *Cell*, **65**, 25-35.

LEONE A, SEEGER RC, HONG CM, HU YY, ARBOLEDA MJ, BRODEUR GM, SLAMON DJ AND STEEG PS. (1993). Evidence for *nm23* RNA overexpression, DNA amplification and mutation in aggressive childhood neuroblastoma. *Oncogene*, **8**, 835-865.

LEVINE AJ. (1993). The tumor suppressor genes. *Annu. Rev. Biochem.*, **62**, 623-651.

LIOTTA LA, STEEG PS AND STETLER-STEVENSON WG. (1991). Cancer metastasis and angiogenesis: an imbalance of positive and negative regulation. *Cell*, **64**, 327-336.

MACDONALD NJ AND STEEG PS. (1993). Molecular basis of tumor metastasis. *Cancer Surv.*, **16**, 175-199.

MACDONALD NJ, DE LA ROSA A, BENEDICT MA, FREIJE JMP, KRUTSCH H AND STEEG PS. (1993). A serine phosphorylation of nm23, and not its nucleoside diphosphate kinase activity, correlates with suppression of tumor metastatic potential. *J. Biol. Chem.*, **268**, 25780-25789.

MORSE HG AND MOORE GE. (1993). Cytogenetic homogeneity in eight independent sites in a case of malignant melanoma. *Cancer Genet. Cytogenet.*, **69**, 108-112.

OKABE-KADO J, KASUKABE T, HONMA Y, HAYASHI M, HENZEL WJ AND HOZUMI M. (1992). Identity of a differentiation inhibiting factor for mouse myeloid leukaemia cells with nm23/nucleoside diphosphate kinase. *Biochem. Biophys. Res. Commun.*, **182**, 987-994.

ORMEROD EJ, EVERETT CA AND HART IR. (1986). Enhanced experimental metastatic capacity of a human tumor line following treatment with 5-azacytidine. *Cancer Res.*, **46**, 884-890.

PARKER C AND SHERBET GV. (1992). Modulators of intracellular $Ca^{2+}$ and the calmodulin inhibitor W-7 alter the expression of metastasis-associated genes MTS1 and NM23 in metastatic variants of the B16 murine melanoma. *Melanoma Res.*, **2**, 337-343.

POSTEL EH, BERBERICH SJ, FLINT SJ AND FERRONE CA. (1993). Human c-*myc* transcription factor PuF identified as nm23-H2 nucleoside diphosphate kinase, a candidate suppressor of tumor metastasis. *Science*, **261**, 478-480.

RADINSKY R, WEISBERG HZ, STAROSELSKY AN AND FIDLER IJ. (1992). Expression level of the *nm23* gene in clonal populations of metastatic murine and human neoplasms. *Cancer Res.*, **52**, 5808-5814.

SASTRE-GARAU X, LACOMBE ML, JOUVE M, VÉRON M AND MAGDELÉNAT H. (1992). Nucleoside diphosphate kinase/nm23 expression in breast cancer: lack of correlation with lymph-node metastasis. *Int. J. Cancer*, **50**, 533-538.

SAWAN A, LASCU I, VÉRON M, ANDERSON JJ, WRIGHT C, HORNE CHW AND ANGUS B. (1994). NDP-K/nm23 expression in human breast cancer in relation to relapse, survival, and other prognostic factors: an immunohistochemical study. *J. Pathol.*, **172**, 27-34.

STEEG PS, BEVILACQUA G, KOPPER L, THORGEIRSSON UP, TALMADGE JE, LIOTTA LA AND SOBEL ME. (1988). Evidence for a novel gene associated with low tumor metastatic potential. *J. Natl Cancer Inst.*, **80**, 200-204.

TOKUNAGA Y, URANO T, FURUKAWA K, KONDO H, KANEMATSU T AND SHIKU H.(1993). Reduced expression of nm23-H1, but not of nm23-H2, is concordant with the frequency of lymph node metastases of human breast cancer. *Int. J. Cancer*, **55**, 66-71.

WARBURTON MJ, MITCHELL D, ORMEROD EJ AND RUDLAND PS. (1982). Distribution of myoepithelial cells and basement membrane proteins in the resting, pregnant, lactating, and involuting rat mammary gland. *J. Histochem. Cytochem.*, **30**, 667-676.

XERRI L, GROB JJ, BATTYANI Z, GOUVERNET J, HASSOUN J AND BONERANDI JJ. (1994). *NM23* expression in metastasis of malignant melanoma is a predictive prognostic parameter correlated with survival. *Br. J. Cancer*, **70**, 1224-1228.